# Genomic alterations in normal breast tissues preceding breast cancer diagnosis

**DOI:** 10.1186/s13058-025-02018-5

**Published:** 2025-04-22

**Authors:** Jiawei Dai, Mariya Rozenblit, Xiaoyue Li, Naing Lin Shan, Yueyue Wang, Shrikant Mane, Michal Marczyk, Lajos Pusztai

**Affiliations:** 1 Yale Cancer Center, Yale School of Medicine, Suite 120, Rm 133, 300 George Street, New Haven, CT 06511 USA; 2 Yale Center for Genome Analysis, West Haven, CT USA; 3 Department of Data Mining and Engineering, Silesian University of Technology, Gliwice, Poland

**Keywords:** Breast cancer precursors, Whole exome sequencing, Early tumorigenesis, Mutation signatures, Immune polymorphisms

## Abstract

**Background:**

Normal breast tissues adjacent to cancer often harbor many of the same genomic alterations as the cancer itself. However, it remains unclear whether histologically normal breast tissues carry genomic changes related to cancer development years before a cancer diagnosis.

**Methods:**

Whole exome sequencing was performed to examine germline and somatic alterations in histologically normal breast tissues from women who subsequently developed breast cancer (*n* = 79, pre-diagnosis tissues) and compared these with results from breast tissues of women who did not (*n* = 81). No patient had germline mutations in cancer predisposition genes.

**Results:**

The pre-diagnosis tissues had significantly more high functional impact germline variants per sample than the healthy controls (*P* = 0.034), 36.5% of affected genes were cancer hallmark genes, among these 62.4% were involved with evading growth suppressors and 5.7% with genome instability. The average number of somatic mutations were similar between the two cohorts. Mutation signature analysis revealed COSMIC signatures 3 (associated with impaired homologous recombination) as a dominant signature more frequent in pre-diagnosis tissues. At gene and variant level, nine common germline polymorphisms in two immune regulatory genes, *FCGBP* and *TPSBP2*, and along with three somatic mutations in *F13A1*, *FRY* and *TMLHE*, were significantly more frequently mutated in the pre-diagnosis samples.

**Conclusions:**

Individuals who develop breast cancer have a higher germline variant burden in normal breast tissues leading to subtle deficiencies in DNA repair that in the context of other germline and somatic mutations could facilitate malignant transformation.

**Supplementary Information:**

The online version contains supplementary material available at 10.1186/s13058-025-02018-5.

## Background

The lifetime probability of being diagnosed with some type of invasive cancer is around 40% for women in the USA, and the most commonly diagnosed malignancy is breast cancer with over 300,000 new cases each year corresponding to a one in eight life time risk [1]. Breast cancer also accounts for one in four cancers in women worldwide with the highest incidence in North America and Europe, and the lowest in South-Central Asia and Africa [2]. Over 90% of breast cancers develop in women with no detectable germline mutation in any high penetrance cancer predisposing genes. However, family history of breast cancer in first degree relatives is a significant risk factor even in the absence of high penetrance germline mutations but genome wide association studies (GWAS) indicate a highly polygenic mode of inheritance. Large GWAS studies identified hundreds of common variants individually associated with small increased (or decreased) risk of breast cancer, with odds ratios (OR) between 0.85 to 1.20 [3], but even when these variants are combined into polygenic risk scores (PRS), they only explain a fraction of heredity. Including family history in PRS-based risk prediction models improves prediction accuracy indicating that yet unknown genetic factors contribute to familial risk [3, 4]. The single most important risk factor for breast cancer is age, with risk increasing substantially after age 45 [5]. This is attributed to age-related accumulation of somatic mutations and epigenetic changes, but the rate of accumulation is influenced by both inherited and environmental factors [6, 7].

Malignant transformation requires alterations in many cellular metabolic and regulatory processes that are collectively described as the hallmarks of cancer [8]. Consistent with gradual accumulation of somatic mutations in cancer relevant genes in normal tissues over lifetime, several studies detected cancer associated molecular changes in cancer-adjacent normal tissues [9–12]. These results suggest that histologically normal tissues can already harbor some of the hallmarks of cancer but have not yet reached a “tipping point” for malignant transformation. The high life-time risk of breast cancer indicates that this stepwise transformation process frequently reaches the critical level for full malignant transformation in breast epithelial cells in women.

The goal of our study was to examine genomic alterations in histologically normal breast tissues of women who several years later developed invasive or non-invasive breast cancer and compare the genomic architecture of these tissues with normal breast tissues from women who have not developed breast cancer. We obtained the tissues from The Susan G. Komen for the Cure Tissue Bank at the Indiana University Simon Cancer Center [13], and used whole exome sequencing (WES) to asses DNA sequence alterations. Our analysis focused on germline and somatic variants with high predicted functional impact (HFI) on protein function and on mutation signatures that could inform about the etiology of the mutagenic process.

## Methods

### Breast tissue samples

Breast core needle biopsy tissues from 160 healthy women at the Susan G. Komen Tissue Bank (https://komentissuebank.iu.edu/) [13] were requested for this study, with informed consent already obtained. The biopsies were collected as part of the Susan G. Komen annual drive when healthy women volunteer to undergo biopsies to donate healthy breast tissue. The biopsies contained normal breast epithelial cells without morphologic abnormalities and associated fat and stroma. The samples were selected to include 79 participants who during the annual follow-up with Susan G. Komen self-reported a subsequent diagnosis of invasive or non-invasive breast cancer that occurred after tissue submission; these cases were classified as pre-diagnosis normal tissues. The other 81 samples included tissues from women who had no cancer diagnosis at the last follow-up, and were classified as healthy control samples. Pre-diagnosis cases and healthy controls were matched by age, body mass index (BMI), Tyrer-Cuzick lifetime risk score, ethnicity, menopausal status, and family history of cancer. None of the participants harbored known germline mutations that predispose to cancer. No Health Insurance Portability and Accountability Act of 1996 (HIPAA) protected information was provided with any of the samples, and therefore this tissue analysis study was exempt from Institutional Review Board (IRB) approval.

### DNA extraction and library Preparation

DNA was extracted using the Chemagic DNA Tissue 100 mg Kit H24 (Revvity, cat no.: CMG-1207). The tissue was lysed in the Lysis buffer/Proteinase K mix at 56 degC. The lysate was prepared and extracted on the Chemagic 360 Instrument (Revvity) according to manufacturer’s protocol. Fragmentation of DNA was accomplished via sonication (Covaris). Fragmented DNA was quality checked using the 4200 TapeStation (Agilent) and High Sensitivity D1000 reagents (Agilent, cat. #5067–5584, 5067–5585). Whole exome libraries were prepared using KAPA HyperPrep kits (Roche, cat. #07962363001) with an input of 1000ng DNA. Libraries were quantified using Quant-IT dsDNA Broad Range assay kit (Thermo Fisher, cat. #Q33130) and multiplexed in pools of 8. Library pools were hybridized and captured using IDT xGen Exome Hyb Panel v2 (IDT, cat. #10005153) and IDT xGen Hybridization and Wash kit (IDT, cat. #1080584). Final libraries were quantified via qPCR on the LightCycler 480 II (Roche Diagnostics) using the KAPA Library Quantification Kit (Roche Diagnostics, cat. #07960298001).

### Whole exome sequencing and data processing

Sample concentrations were normalized to 2nM and loaded onto Illumina NovaSeq X Plus flow cell at a concentration that yields at least 600Gbp data per lane. The loading concentration for WES libraries was optimized to maximize both well occupancy and unique read output while limiting duplicates associated with patterned flow cell technology. Samples were sequenced at a target coverage of 150x using 101 bp paired-end sequencing reads according to Illumina protocols. The 10 bp indexes were read during additional sequencing reads that automatically followed the completion of read 1. A positive control (bacteriophage Phi X library) provided by Illumina was spiked into every lane at a concentration of 1% to monitor sequencing quality in real time. Signal intensities were converted to individual base calls during a run using the system’s Real Time Analysis (RTA) software. Sample de-multiplexing and alignment to the human genome - were performed using Illumina’s CASAVA 1.8.2 software suite. The error rate for all sample data was less than 2%.

A flow diagram illustrating somatic and germline variant calling pipeline is shown in Additional file 2: Fig. S1. The raw sequencing reads in FASTQ format were processed using Trim Galore (version 0.6.10) with default settings for quality and adapter trimming [14]. The cleaned reads were then aligned to the human reference genome GRCh38 (hg38) using the Burrows–Wheeler aligner (BWA, version 0.7.17-r1188) [15]. Subsequently, we followed the workflows of the Genome Analysis Toolkit (GATK, version 4.4.0.0) to detect both somatic and germline variants [16]. Duplicated reads were marked and removed using the GATK’s Picard tool. Base quality score recalibration was performed using the GATK’s BaseRecalibrator and ApplyBQSR tools. Since we had no matching germline DNA from blood, for somatic variant calling we used the GATK’s Mutect2 tool in tumor-only mode. This mode uses a pre-assembled Panel of Normals (PON), constructed from hundreds to thousands of normal samples, to exclude germline variants [17]. Additionally, the resource gnomAD, which contains population allele frequencies of both common and rare variants, was used to further exclude germline variants. This process was followed by variant filtering with the GATK’s FilterMutectCalls tool to reduce false positives and enhance call accuracy. Previous studies have indicated that the GATK’s HaplotypeCaller tool can detect germline variants even in tumor-only sequencing [18, 19]. Therefore, we employed it for germline variant calling in normal breast tissues from pre-diagnosis cases and healthy controls. Germline variants were defined as variants that vary against the reference as determined by HaplotypeCaller, with subsequent filtering by the GATK’s CNNScoreVariants and FilterVariantTranches tools. CNNScoreVariants annotated the variants used a pre-trained convolutional neural network model, and FilterVariantTranches applied tranche filtering based on scores from CNNScoreVariants and common variants in the resource files including HapMap, Mills and 1000G, to improve the accuracy and reliability of germline variant identification. We further required the somatic variants to have an Allelic Depth (AD) > 5 for the alternative allele and a variant allele frequency (VAF) > 5%. For germline variants the AD had to be > 5 and VAF > 20%.

### Ancestry inferred from genotypes

The ancestry of participants was inferred using the genotype-based tool GrafPop (version 1.0) [20, 21]. We used the germline variants as input for GrafPop, and default parameters were employed. Genetic distances from each participant to reference populations were calculated, and then the ancestral proportions of participants (European, African, and East Asian) were estimated.

### High functional impact variants

Variants were annotated with variant type, MetaSVM deleteriousness and ClinVar pathogenicity (version 2022-12-31) using ANNOVAR (version 2020-06-08) [22]. A variant was classified high functional impact if it was classified as Deleterious by MetaSVM with a score above 0, or if it was designated as Pathogenic/Likely Pathogenic in ClinVar, or if it resulted in frameshift, start loss, stop gain, or stop loss variants, or if the variant was already annotated as high-confidence loss-of-function in the gnomAD database (version 2.1.1, https://gnomad.broadinstitute.org/).

Variants were also assigned into cancer hallmark categories if the associated genes belonged to biological processes that comprise the hallmarks of cancer (https://cancerhallmarks.com/). Additionally, variants were classified into mutation significance tiers based on Catalogue of Somatic Mutations In Cancer (COSMIC, https://cancer.sanger.ac.uk/cosmic, version 101) Cancer Mutation Census (Additional file 1: Table S1 and S2). Tier 1 mutations were strongly associated with cancer; tier 2 mutations were of medium significance for cancer relevance and potentially associated; tier 3 variants were of low significance, with minimal impact on cancer. ‘Other’ included mutations with no predicted significance.

### Mutational signature analysis

Different mutational processes generate unique combinations of nucleotide alterations that can inform about the mutagenic processes. We performed somatic mutation signature analysis using the non-negative matrix factorization (NMF) algorithm SignatureAnalyzer (version 0.0.8) [23, 24]. The detected signatures were compared to the COSMIC signatures to determine cosine similarity to 30 canonical signatures. SignatureAnalyzer was used to infer activity level for each detected signature (i.e., the estimated number of mutations associated with each signature). The activity level quantifies the contribution weight of each mutational signature to all mutations in an individual sample.

### Expression data of normal breast and invasive breast cancer

mRNA expression data of normal and cancerous breast tissues was acquired from The Cancer Genome Atlas (TCGA, https://portal.gdc.cancer.gov/). The Transcripts per Million (TPM) raw read counts for 110 normal samples and 1,113 cancerous samples were downloaded and log_2_(TPM + 1) transformed for further analysis. We also downloaded two additional microarray expression datasets, E-GEOD-70951 and E-GEOD-76250, from ArrayExpress (https://www.ebi.ac.uk/arrayexpress/), which included 148 and 33 paired samples of breast cancer and normal breast tissues, respectively. The microarray data were quantile normalized and log_2_-transformed.

Core cell types associated with specific genes in tissues were derived from the Human Protein Atlas (https://www.proteinatlas.org/) [25]. This classification identifies genes that demonstrate enriched specificity across various cell types within a single or multiple tissues, or those predominantly expressed in a core cell type present in numerous tissues. The single-cell RNA expression levels of the genes in breast tissues were sourced from the Breast Cancer Atlas of Single Cell portal (https://singlecell.broadinstitute.org/single_cell/study/SCP1039) [26].

### Statistical analysis

Two-sample t-test was conducted to analyze differences of participant characteristics in continuous variables, including age, BMI, Tyrer-Cuzick lifetime risk score and follow-up time, between groups. For categorical variables, such as ethnicity, menopausal status and family history of cancer, Fisher’s exact test was used to test for significance. Fisher’s exact test was performed to detect differentially mutated genes/variants. Genes with variants were considered mutated genes. Only genes/variants that were mutated in at least five samples were considered, to avoid bias from mutations in very few samples and to increase statistical effectiveness. *P* values were adjusted for false positive rate (FDR) using the Benjamini-Hochberg method. Genes or variants with FDR below 0.05 were considered significant. The population allele frequencies of differentially mutated variants in females were obtained from the gnomAD database. Shapiro-Wilk test was conducted to assess the normality of the number of mutations and gene expression. Since the Shapiro-Wilk test resulted in a *P* value of 0.4843 for the total number of mutations, confirming their normal distribution, the number of mutations was compared using two-sample t-test. However, as the Shapiro-Wilk test *P* values were less than 0.05 for most of the genes, the mRNA expression of these genes was compared using the Wilcoxon rank sum test, and the microarray expression was compared using the Wilcoxon signed rank test. Statistical significance was defined as *P* < 0.05. All analyses and visualizations were performed using R (version 4.3.1).

## Results

### Study participant characteristics

The study participant characteristics of pre-diagnosis breast tissues (*n* = 79) and the tissues from women who have not developed breast cancer (i.e. healthy controls, *n* = 81) are presented in Table 1. The self-reported ethnicity of participants matched closely the genotype-based ancestry (Additional file 2: Fig. S2). There were no significant differences in age, race, length of follow-up since tissue donation, BMI, Tyrer-Cuzick lifetime risk score, menopausal status, and family history of cancer between the cohorts. Among the pre-diagnosis samples, the average time from tissue donation to breast cancer diagnosis was 4.7 years (range: 0–11), among the healthy controls the average time from tissue donation to last follow up was 6.1 years (range: 0–14) (*P* = 0.028).

**Table 1:** Study participant characteristics

	Healthy controls (*n* = 81)	Pre-diagnosis tissues (*n* = 79)	*P* value
Age, mean (range)	53.9 (29–73)	53.6 (28–74)	0.837^a^
BMI, mean (SD)	30.4 (6.3)	30.6 (6.5)	0.904^a^
Tyrer-Cuzick lifetime risk score, mean (SD)	12.7 (6.7)	13.4 (9.7)	0.594^a^
Self-reported ethnicity, n (%)			
Black	17 (21.0%)	17 (21.5%)	1^b^
White	62 (76.5%)	60 (75.9%)	
Others	2 (2.5%)	2 (2.6%)	
Menopausal status, n (%)			
Pre-menopausal	23 (28.4%)	23 (28.8%)	0.476^b^
Post-menopausal	53 (65.4%)	47 (60.0%)	
Others	5 (6.2%)	9 (11.2%)	
Has blood relatives with cancer, n (%)			
Yes	43 (53.1%)	44 (55.0%)	1^b^
No	34 (42.0%)	33 (42.5%)	
	NA (4.9%)	NA (2.5%)	
Time to diagnosis in years, mean (range)	NA	4.7 (0–11)	
Follow-up time from donation year, mean (range)	6.1 (0–14)	NA	

^a^*P* values determined by two-sample t-test

^b^*P* values determined by Fisher’s exact test

### The mutational landscape of breast tissues

**Fig. 1:**
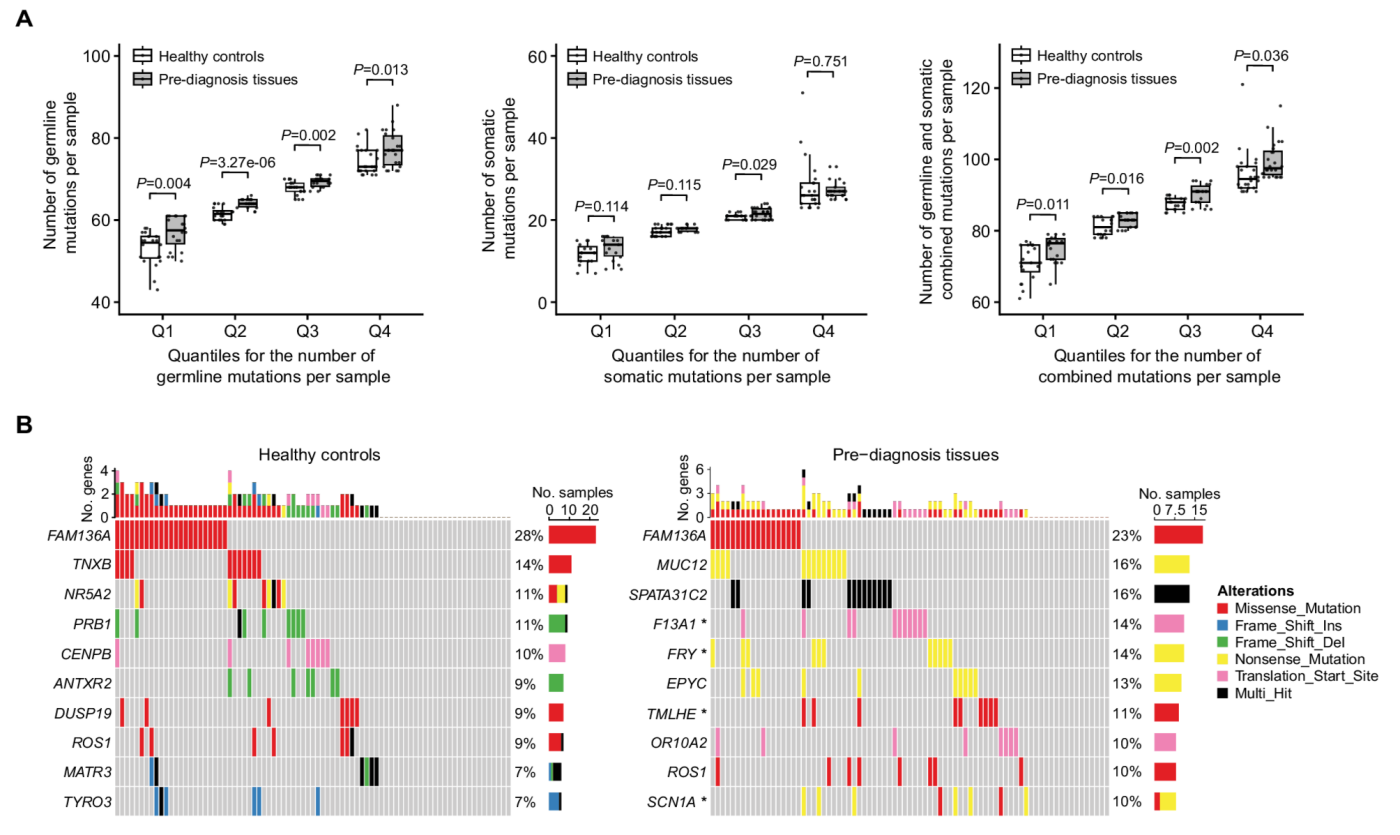
Somatic mutation landscape in pre-diagnosis tissues versus healthy controls. **A** Comparison of the number of mutations per sample categorized by source (germline, somatic, or combined) between the two groups, across four quartiles. **B** Oncoplots of genes that are most frequently affected by somatic mutations in the two different cohorts. Percent numbers indicate the fraction of cases affected. Asterisks indicate genes with significantly different mutation frequencies between the groups

Overall, the pre-diagnosis tissues had significantly more HFI variants per sample than breast tissues from healthy controls. As detailed in the Materials and Methods, the sequencing coverage was consistent across the two cohorts, confirming that the observed differences are not due to sequencing artifacts. When variants were compared between the cohorts separately based on somatic versus germline origin, there were significantly higher number of germline variants in the pre-diagnosis samples compared to breast tissues from healthy controls (*P* = 0.034). The average numbers of somatic mutations per sample were similar between the two cohorts. This trend was observed across all quartiles of germline variant and mutation burden, with quartiles calculated independently for each cohort. (Fig. 1A). Additional file 1: Table S1 and S2 list all genes effected by HFI germline variants and somatic mutations for each sample. These results suggest that individuals who develop breast cancer have a higher variant burden in normal breast tissues and most of these variants are of germline origin. Variants were distributed across all cancer hallmark genes with no variant enrichment (Additional file 1: Table S3).

At gene level, there was no statistically significant difference in the frequencies of genes harboring HFI germline variants in pre-diagnosis compared to healthy control samples despite the overall higher germline variant burden. 36.5% of genes affected by HFI germline variants were cancer hallmark genes, among these 62.4% were involved with evading growth suppressors and 5.7% with genome instability. When somatic mutations were examined, 38.6% of genes affected were cancer hallmark genes, among these 40.7% were involved with evading growth suppressors and 9.5% with genome instability. We also identified four genes that were significantly more frequently somatically mutated in pre-diagnosis tissues that eventually gave rise to cancer (Fig. 1B). These genes included Transglutaminase A Chain (*F13A1*) in 14% (versus 1% in controls), FRY Microtubule Binding Protein (*FRY*) affected in 14% (versus 0% in control), Trimethyllysine Hydroxylase, Epsilon (*TMLHE*) in 11% (versus 0% in controls), and Voltage-gated Sodium Channel Type I alpha Subunit (*SCN1A*) in 10% (versus 0% in controls) (Additional file 2: Fig. S3). *SCN1A* harbored five different variants in different individuals, unlike the other genes that had recurrent variants. No gene was more frequently affected by somatic mutation in the healthy control tissues compared to the pre-diagnosis cohort.

Figure 1B shows the top 10 most frequent somatically mutated genes in the two cohorts. We also observed many somatic nonsense mutations in the top 10 mutated genes in pre-diagnosis tissues. Nonsense mutations lead to truncated proteins often with high impact on function, we therefore compared the number of nonsense mutations between the two cohorts. There was a trend toward more somatic nonsense mutations in pre-diagnosis tissues, with an average of 4.5 (range: 0–11), compared to an average of 3.8 (range: 0–16) in healthy controls (*P* = 0.086). No difference was found in the number of germline nonsense mutations between the two groups (*P* = 0.275).

**Fig. 2:**
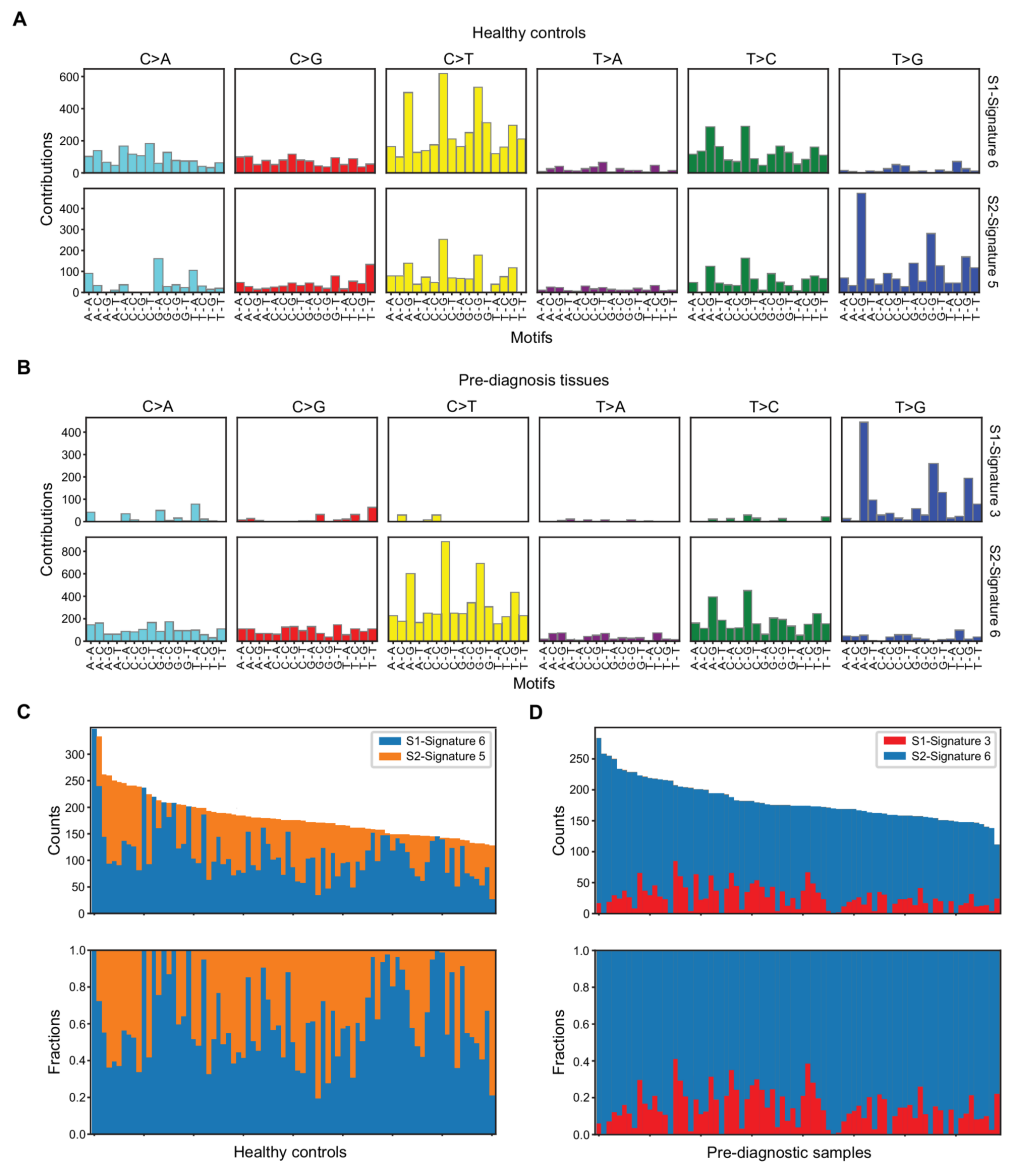
COSMIC mutation signatures identified in healthy control and pre-diagnosis breast tissues. **A** Mutational signature profiles representing 96 nucleotide combinations in healthy controls. **B** Mutational signature profiles in pre-diagnosis tissues. **C** Counts and fractions of COSMIC signatures 5 and 6 in healthy control breast tissues. **D** Counts and fractions of COSMIC signatures 3 and 6 in pre-diagnosis tissues

Mutation signature analysis of healthy control tissues detected COSMIC signatures 5 and 6 with cosine similarities of 0.57 and 0.85, respectively (Fig. 2A and C). The etiology of signature 5 is unknown, but signature 6 is attributed to defective DNA mismatch repair and is also found in microsatellite unstable tumors. In the pre-diagnosis samples, COSMIC signatures 3 and 6 were detected, with cosine similarities of 0.32 and 0.84 (Fig. 2B and D). Signature 3 is associated with failure of DNA double-strand break-repair by homologous recombination. We also observed slightly more genome instability hallmark genes affected by HFI variants in pre-diagnosis tissues with higher activity levels of signatures 3. For signature 3, samples with activity above the median had an average of 0.4 germline variants and 0.7 somatic mutations in genome instability genes whereas samples below the median had an average of 0.2 germline variants and 0.6 somatic mutations. For signature 6, both high and low activity samples had an average of 0.3 germline variants and 0.7 somatic mutations in DNA repair genes. These findings suggest that germline variants in genes that regulate genome stability could affect mutation signature patterns in normal tissues, and subsequent cancer risk.

### High functional impact variants more frequently mutated in pre-diagnosis breast tissues

**Fig. 3:**
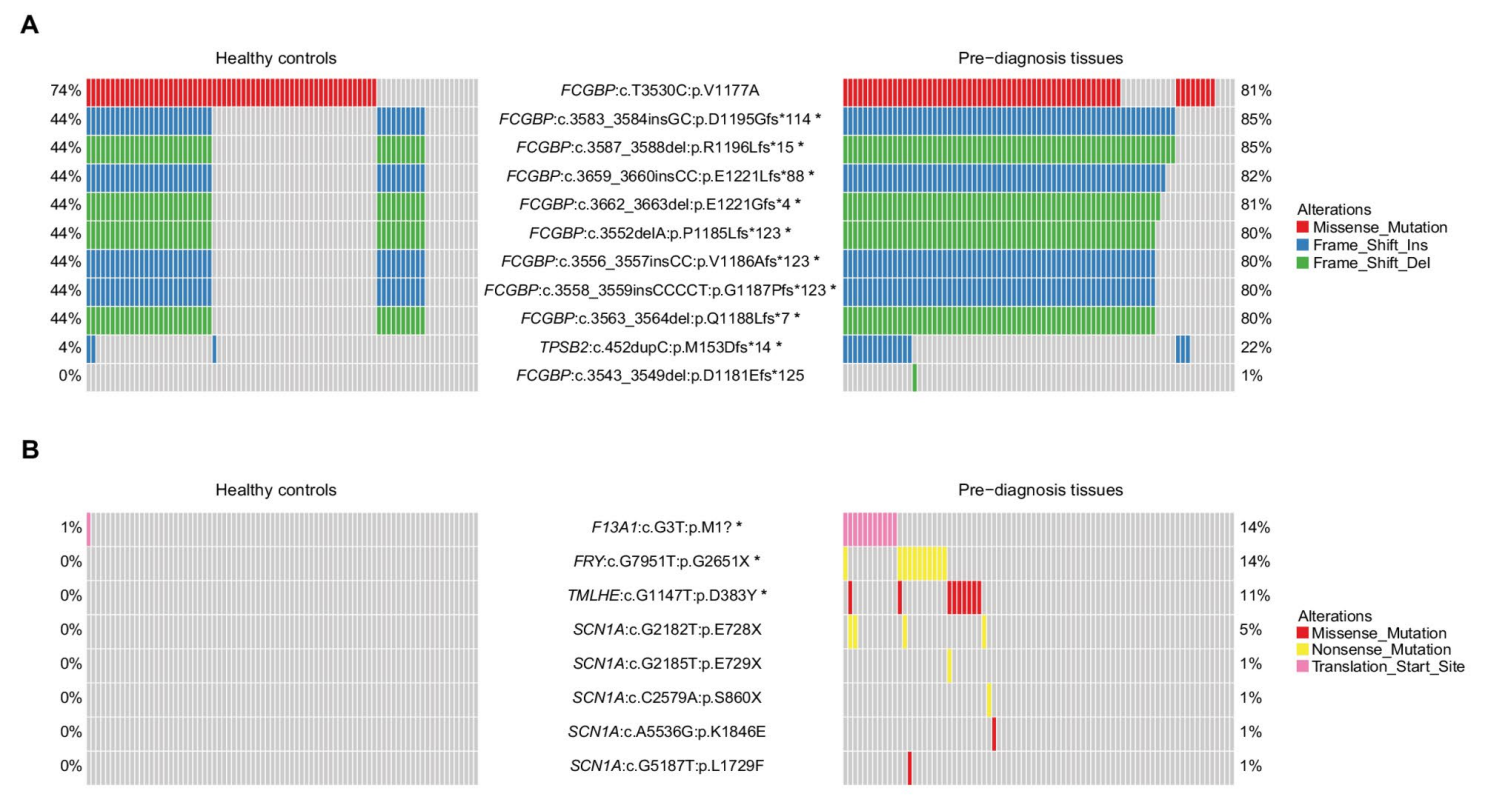
Variant level differences between pre-diagnosis breast tissues and healthy controls. **A** Oncoplots for germline variant alleles for the *FCGBP* and *TPSB2* genes in the two groups. **B** Oncoplots for somatic mutations in the four significantly differently affected genes in the two groups. Asterisks indicate variants with significantly different mutation frequencies between the groups

**Fig. 4:**
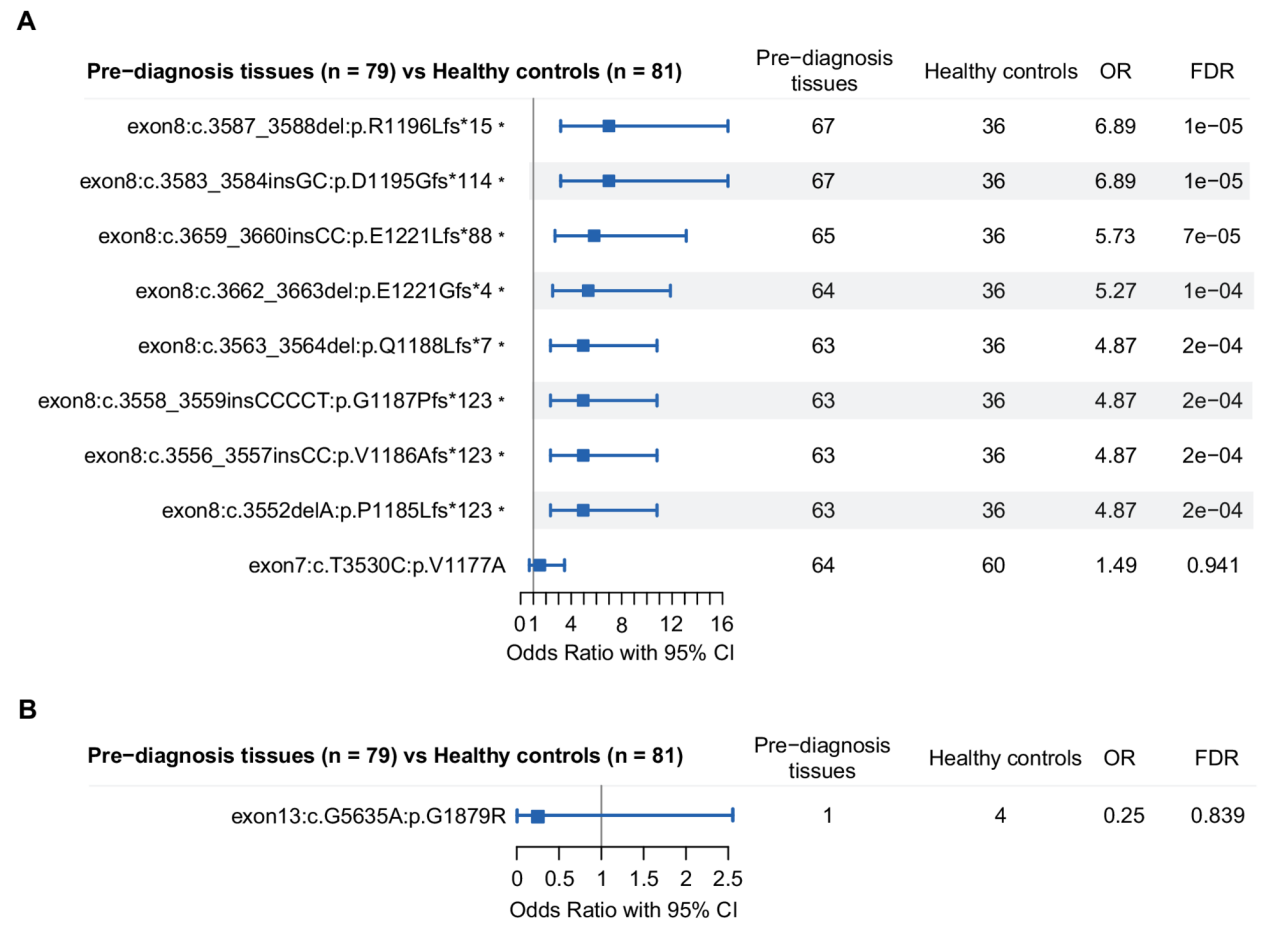
Germline variant alleles and Somatic variant in the *FCGBP* gene. **A** Germline variant allele frequencies. **B** Somatic variant allele frequencies. Asterisks indicate variants that are more frequently found in pre-diagnosis tissues compared to healthy controls

When we compared HFI germline variant allele frequencies, nine alleles were more frequently mutated in pre-diagnosis cohort compared to healthy controls (OR > 1.0, FDR < 0.05, Fig. 3A). All of them were common polymorphisms and none were previously linked to breast cancer risk in genome-wide association studies [27–29]. Eight of these variants were frameshift mutations in exon 8 of the Fc-Gamma Binding Protein (*FCGBP*) gene (Fig. 4, Additional file 2: Fig. S4A). The most common variant was c.3587_3588del: p.R1196Lfs*15 detected in 85% of pre-diagnosis tissues and 44% of healthy controls (OR = 6.89; FDR = 1e-05). The population allele frequencies of these eight variants in the gnomAD database confirmed high allele frequencies ranging from 0.764 to 0.971, with uniform frequencies across ancestry groups (Additional file 1: Table S4). The germline variant, c.452dupC: p.M153Dfs*14 in exon 4 of the Tryptase Beta-2 (*TPSB2*) gene, also showed significantly higher frequency in pre-diagnosis tissues compared to healthy controls (22% vs. 4%; OR = 7.05; FDR = 0.021) (Fig. 3A, Additional file 2: Fig. S4B). The population allele frequency of this variant is lower (0.211), but still in the range of common polymorphisms (i.e. > 0.01) among individuals with African and European ancestry, but much lower among East (0.00099) and South (0.084) Asian women (Additional file 1: Table S4).

When we compared somatic mutation allele frequencies, we found three mutations that were significantly more frequent in pre-diagnosis tissues (Fig. 3B, Additional file 2: Fig. S4C-F). The c.G3T: p.M1? mutation in the *F13A1* gene occurred in 14% versus 1% in pre-diagnosis and healthy control samples (OR = 12.79; FDR = 0.035). The c.G7951T: p.G2651X mutation in the *FRY* gene and the c.G1147T: p.D383Y mutation in the *TMLHE* gene were found in 14% (FDR = 0.014) and 11% (FDR = 0.033) of pre-diagnosis tissues, respectively, with no mutations seen in the healthy controls. *SCN1A* gene was found to be significantly more mutated in pre-diagnosis samples. However, none of the five mutations demonstrated a significant difference between the two cohorts (Fig. 3B). Germline population allele frequencies of these three somatic mutations in the *F13A1*, *FRY*, and *TMLHE* genes were very low, ranging from 0.000045 to 0.0016, and similar across ancestry groups. Interestingly, we found two somatic mutations, *PIK3CA*:c.G1624A: p.E542K and *PIK3CA*:c.A3140G: p.H1047R, which were of high significance according to COSMIC, and two others, *AKT1*:c.G49A: p.E17K and *NCOR1*:c.334delG: p.E112Nfs*18, of low significance in pre-diagnosis tissues. In contrast, no somatic mutations of cancer significance were identified in healthy controls (Additional file 1: Table S2).

### Expression levels of the *FCGBP*, *TPSB2*, *F13A1*, *FRY*, *TMLHE*, and *SCN1A* genes in breast tissues and in different cell types in the breast

**Fig. 5:**
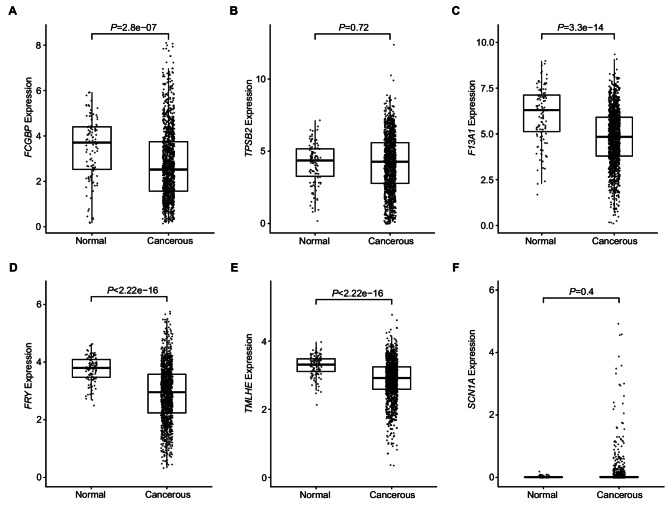
mRNA expression levels of the six genes in normal and cancerous TCGA breast tissues. A *FCGBP*, B *TPSB2*, C *F13A1*, D *FRY*, E *TMLHE*, F *SCN1A*. *P* values were from Wilcoxon rank sum test

We examined mRNA expression levels of the six genes that were more frequently affected by HFI germline variants or somatic mutations in breast tissues that subsequently developed invasive or non-invasive cancer. In the TCGA, five of the six genes showed generally high mRNA expression levels in both normal and cancer tissues, except *SCN1A* that had very low expression (Fig. 5). The low/absent expression of *SCN1A* is consistent with its restricted expression in neural and muscle tissues. The *FCGBP*, *F13A1*, *FRY*, and *TMLHE* genes had significantly higher expression in normal tissues than in cancer (*P* = 2.8e-07, *P* = 3.3e-14, *P* < 2.22e-16 and *P* < 2.22e-16, respectively). *TPSB2* showed similar expression in normal and cancer tissues. Similar results for the *FCGBP*, *F13A1*, *FRY*, and *TMLHE* genes were observed in the two additional microarray expression datasets, E-GEOD-70,951 and E-GEOD-76,250 (Additional file 2: Fig. S5). *TPSB2* was not found in any microarray datasets. Additionally, we compared the descending rankings of the six genes relative to the entire transcriptome based on their mRNA expression levels in the TCGA (Additional file 1: Table S5). Among 19,938 human genes, *FCGBP*, *TPSB2*, *F13A1*, *FRY*, and *TMLHE* exhibited moderately high expression levels in the breast tissues. Compared to normal breast tissues, the expression levels of *FCGBP*, *F13A1*, *FRY*, and *TMLHE* were ranked lower in cancerous tissues (*P* = 2.3e-06, *P* = 8.1e-15, *P* < 2.2e-16, and *P* = 4.0e-09 respectively). *SCN1A* showed very low expression compared to other genes, with a slightly higher ranking in cancerous tissues compared to normal breast tissues (*P* = 0.0012).

We also assessed what core cell types express these six genes in various tissues using data from the Human Protein Atlas (Additional file 2: Fig. S6) and single-cell RNA expression results from the Breast Cancer Atlas Single Cell portal (Additional file 2: Fig. S7). *FCGBP* is primarily expressed in macrophages in a range of tissues, including adipose tissues, pancreas, skeletal muscle, and breast. In the single-cell data set, *FCGBP* was enriched in macrophages, monocytes, cycling myeloid cells, and T cells. *TPSB2* is also primarily expressed in macrophages and mast cells in adipose tissues. In single-cell analyses of breast tissues, *TPSB2* exhibited broad expression across cell types. *F13A1* is another macrophage associated gene that is expressed across several tissues, including breast. In single-cell data of breast tissues, it is expressed in cycling myeloid cells, macrophages, monocytes, and cancer-associated fibroblasts (CAFs). In contrast, the other three genes did not show enrichment in specific cell types across various tissue types. However, single-cell data from breast tissues indicated that *FRY* was predominantly expressed in cycling perivascular-like (PVL) cells and endothelial cells. The *TMLHE* gene showed similar expression levels across all cell types in the single-cell data. *SCN1A* showed no expression across breast tissue cells. These results suggest that subtle innate immunity differences mediated through germline polymorphisms in *FCGBP* and *TPSB2* genes or acquired mutations in *F13A1* could influence breast cancer risk.

## Discussion

We examined germline polymorphisms and somatic mutations in histologically normal breast tissues of women who subsequently developed breast cancer and women who have during an average follow-up time of 6 years. High functional impact germline polymorphisms and acquired somatic mutations were frequently detected in cancer-relevant genes in both cohorts, however the pre-diagnosis tissues on average had significantly more HFI germline variants per sample. Several previous studies have demonstrated that normal breast tissues adjacent to cancer harbor many of the same genomic alterations that the cancer has [9–11]. We now show that high functional impact protein altering somatic mutations in cancer hallmark genes can also be found in histologically normal breast tissues many years before diagnosis of cancer. Equally importantly many cancer hallmark genes are also affected by germline variants. In pre-diagnosis tissues, of all genes affected by HFI germline polymorphisms 36.5% were cancer hallmark genes, and of the genes altered by somatic mutations 38.6% were cancer hallmark genes. The higher number of germline variants in pre-diagnosis tissues compared to controls suggests that individuals who are born with many individually not cancer-risk conferring variants are more susceptible for breast cancer development than individuals with lower variant burden in these genes. We hypothesize that those with high germline variant burden may require fewer additional somatic alterations to reach the critical level of cellular process disturbance for malignant transformation. This hypothesis is supported by a previous observation that across all cancer types, higher germline variant burden in cancer-relevant genes correlates with earlier onset of cancer, whereas earlier onset cancers have lower somatic mutation burden [6]. The current study is too small, and has too narrow of an age range, to address if germline variant burden correlates with age of onset of breast cancer. Mutation signature analysis suggested a possible mechanism for the higher variant burden observed in the pre-diagnosis cohort, the two signatures, COSMIC 3 and 6, that we detected in these tissues are both related to DNA repair deficiency. However, as the number of somatic variants was similar between the two cohorts, the accumulation of somatic mutations due to subtle DNA repair deficiencies in pre-diagnosis tissues may require a long time to reach a statistically detectable level. These results suggest that subtle deficiencies in DNA repair may exist in normal breast tissues of women who develop breast cancer. Which genes or variants might cause this deficiency is difficult to elucidate because at gene level, different combinations of genes were affected by HFI germline variants in different individuals. The mutation signatures likely represent phenotypic convergence driven by different mechanism in different individuals.

We also noted higher frequencies of nine common germline polymorphisms in two immune regulatory genes, *FCGBP* and *TPSB2* that are primarily expressed in macrophages. Eight of the variants were frame shift variants in the *FCGBP* gene and at least one of these variants were seen in almost all pre-diagnosis tissues compared to less than half of the controls. We also observed the co-occurrence of more than one of these mutations in pre-diagnosis samples (Fisher’s exact test *P* = 1.953e-14). Since these mutations are very consistent across samples, they might all come from the same underlying genomic event, such as a complex chromosomal recombination event or a shared haplotype. The *FCGBP* gene encodes a high molecular weight glycoprotein that binds to the Fc portion of immunoglobulin-G and plays an important role in innate mucosal epithelial defense, but also influences tumor metastasis and tumor immunity [30]. *FCGBP* expression is correlated with higher immune infiltration and better prognosis in various cancer types and its down regulation is associated with an immunosuppressive tumor microenvironment [31–33]. The *TPSB2* gene encodes a serine protease that is the most abundant mediator stored in mast cell granules and plays a central role in activating innate immunity, inflammatory and allergic reactions [34]. Mast cell activity in breast cancer tissues have context dependent immune activating or immune suppressing effect, but in most studies mast cells are associated with poorer prognosis and grater chemotherapy resistance [35]. These findings raise the possibility that some subtle deficiency in innate and adaptive anti-tumor immune surveillance that fails to eradicate transforming cells in breast tissues could contribute to breast cancer development in some women.

Similar to the variable combination of HFI germline polymorphisms, we found that different combinations of genes were affected by somatic mutations in different individuals. However, three highly expressed genes in breast tissues showed significantly higher rates of somatic mutations in pre-diagnosis tissues than in controls. The two most frequently mutated genes *FRY* and *F13A1* were both mutated in 14% of pre-diagnosis cases and almost never in controls, and the *TMLHE* gene was mutated in 11% of cases but not in any of the controls. *FRY* is microtubule associated protein and plays a role in chromosome alignment and stabilizes microtubules during mitosis. A truncated versions in the form of *EEF1DP3*-*FRY* fusion gene was detected in about 7% of breast cancers [36], polymorphisms in *FRY* were also linked to mammary tumor susceptibility in F334 rats [37], and *FRY* conditional knockout mice showed impaired mammary gland development during pregnancy and the forced expression of the gene suppressed breast cancer cell growth [38]. *F13A1* is a component of coagulation factor XIII and functions as a transglutaminase that can also cross-link many proteins involved in tumor growth, wound healing and apoptosis [39]. *F13A1* expression increases proliferation, invasion and migration of oral squamous cell carcinoma cells in vitro [40]. Its role in breast cancer biology is unexplored although coagulation-related gene expression has been associated with prognosis and chemotherapy response [41]. The *TMLHE* gene encodes enzyme that catalyzes the initial rate limiting step in carnitine biosynthesis and is required for histone acetylation and efficient DNA repair [42]. It was also found to be critical for triple negative breast cancer cell growth [43]. These results suggests that although these genes are not classical oncogenes, their altered functions may enable cells to progress towards transformation. The highly variable and individual combinations of somatic mutations and germline protein altering variants hint at many distinct paths through which genomic alterations could converge towards malignant transformation.

Our study has important limitations, in the absence of matching peripheral blood DNA our classification of variants into germline versus somatic origin was based on whitelisting of known polymorphisms, but we recognize that some of our predicted somatic variants could also represent germline variants. Three quarters of our study population were White and germline polymorphisms are less well catalogued in non-White populations in the USA. The median follow-up time for the control group was around 6 years and some of these individuals could develop breast cancer at later time points. Furthermore, we used expression datasets to estimate the impact of mutations in the six genes, which indicated reduced expression levels in tumors. However, future functional studies will be needed to confirm the biological importance of these alterations.

## Conclusions

In summary, our findings add to the growing literature which indicates that histologically normal breast tissues harbor genomic alterations in various cancer hallmark pathways. Our data also suggest that a combination of subtle inter-individual differences in DNA repair fitness and germline polymorphisms that possibly affect innate immunity can “set the scene” for subsequent malignant transformation and breast cancer development. The absence of highly recurrent somatic or germline alterations in the pre-diagnosis tissues also indicate that there are many different paths to malignant transformation enabled by disturbances in different genes at different nodes in the large network of biological pathways that regulate cell growth, invasion, cellular metabolism, and immune escape.

## Electronic supplementary material

Below is the link to the electronic supplementary material.

Supplementary Material 1

Supplementary Material 2

## Data Availability

The dataset generated during the current study is available in the dbGap repository under accession number phs003822.v1.p1 (https://www.ncbi.nlm.nih.gov/projects/gap/cgi-bin/study.cgi?study_id=phs003822.v1.p1).

## Abbreviations

GWAS: Genome wide association studies
OR: Odds ratios
PRS: Polygenic risk scores
WES: Whole exome sequencing
HFI: High functional impact
BMI: Body mass index
HIPAA: Health Insurance Portability and Accountability Act of 1996
IRB: Institutional Review Board
RTA: Real Time Analysis
BWA: Burrows–Wheeler aligner
GATK: Genome Analysis Toolkit
PON: Panel of Normals
AD: Allelic Depth
VAF: variant allele frequency
COSMIC: Catalogue of Somatic Mutations In Cancer
NMF: Non-negative matrix factorization
TCGA: The Cancer Genome Atlas
TPM: Transcripts per Million
FDR: False discovery rate
CAFs: Cancer-associated fibroblasts
PVL: Perivascular-like
