# Supplementary material for: Genomic alterations in normal breast tissues preceding breast cancer diagnosis
Source: Breast Cancer Res. 2025 Apr 22;27:60. doi: 10.1186/s13058-025-02018-5 (PMC12013151; doi:10.1186/s13058-025-02018-5)
Supplement: Supplementary file 2 — Supplementary Material 2 [file 13058_2025_2018_MOESM2_ESM.docx]

**Additional file 2**


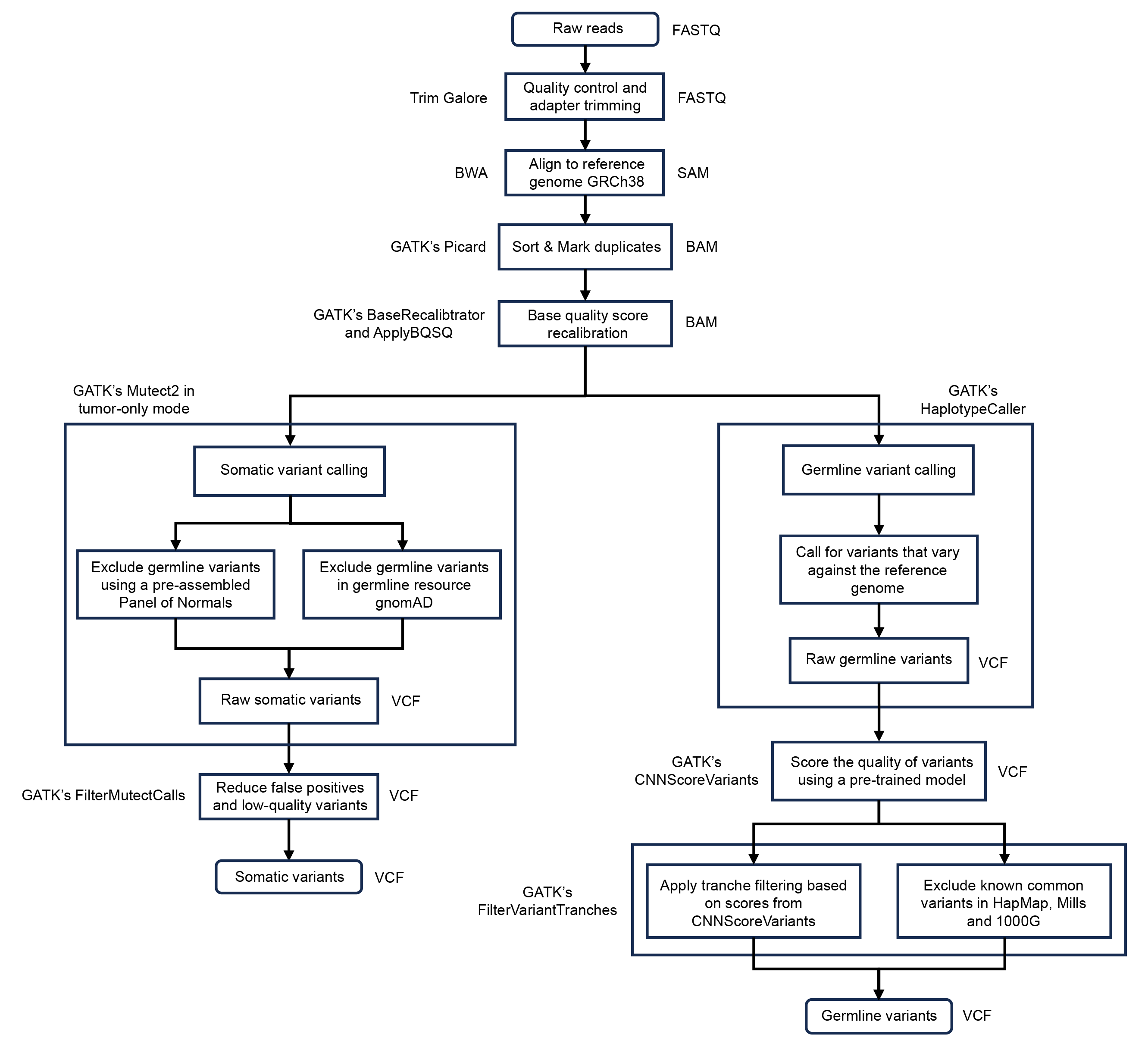


**Fig. S1. Flow diagram for the calling of somatic and germline variants.**

**
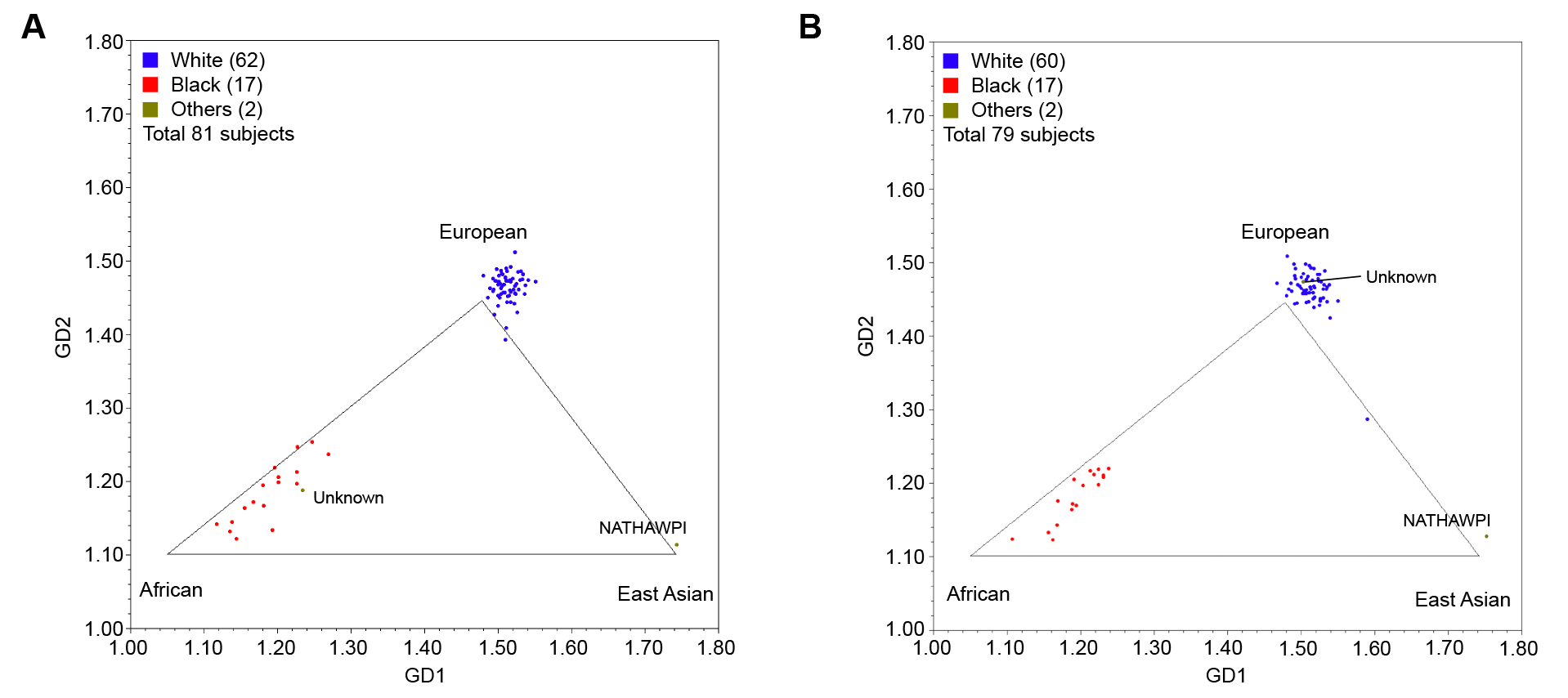
**

**Fig. S2. Subject ancestry inferred from genotypes**. **A** Ancestry of healthy controls. **B** Ancestry of pre-diagnosis tissues. The self-reported ethnicity of the samples is represented in three colors. Categories of ‘Others’ samples are specifically labeled in the figure. Each corner of the triangle represents a different inferred ancestry. A dot closer to a specific corner indicates that the sample is more likely to belong to that ancestry. GD: genetic distance.


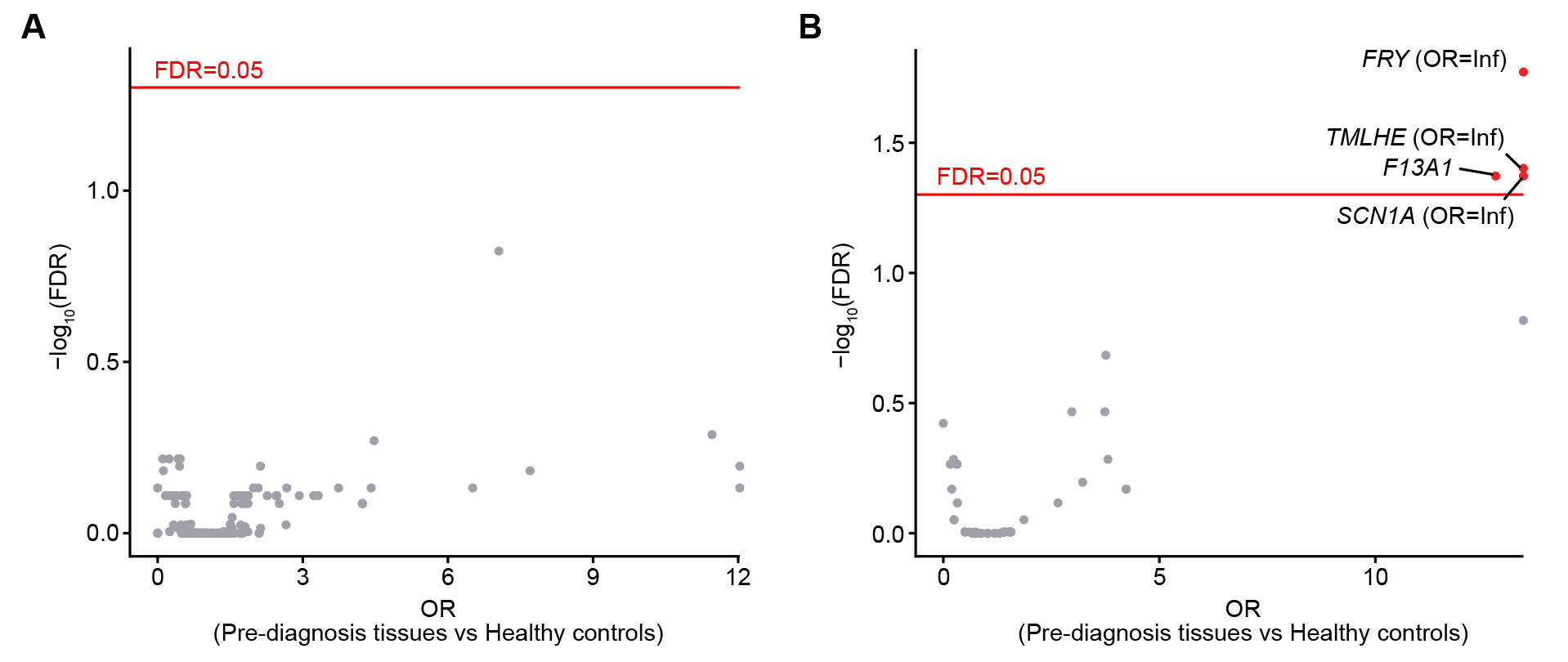


**Fig. S3.** **Differentially mutated genes from** **A** germline, and **B** somatic origins between pre-diagnosis breast tissues and healthy controls.


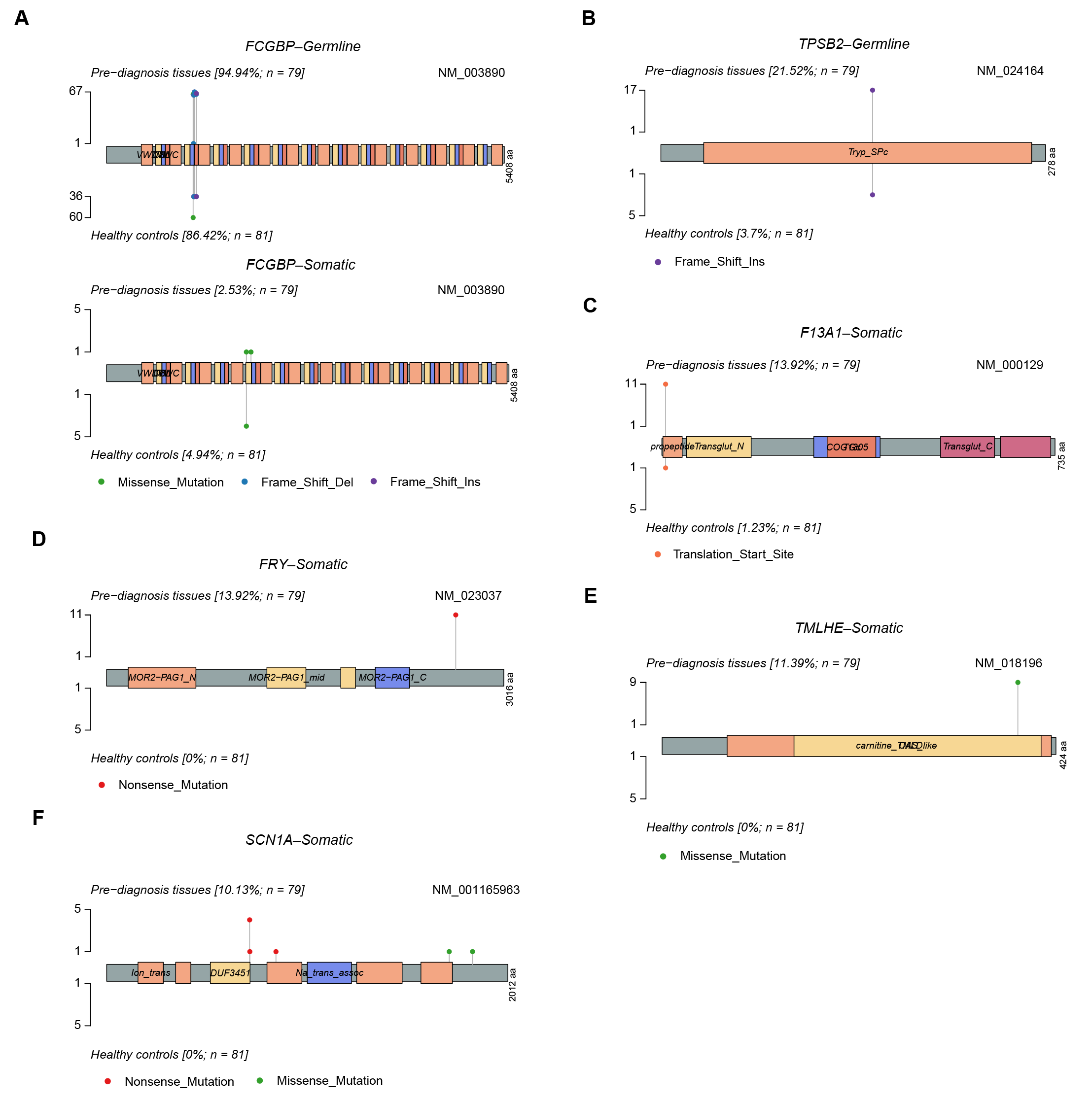


**Fig. S4.** **Mutations in the six genes presented as lollipop plots, categorized by variant type.** **A** *FCGBP*, **B** *TPSB2*, **C** *F13A1*, **D** *FRY*, **E** *TMLHE*, **F** *SCN1A*. Lollipops without germline or somatic mutations are not shown.

**
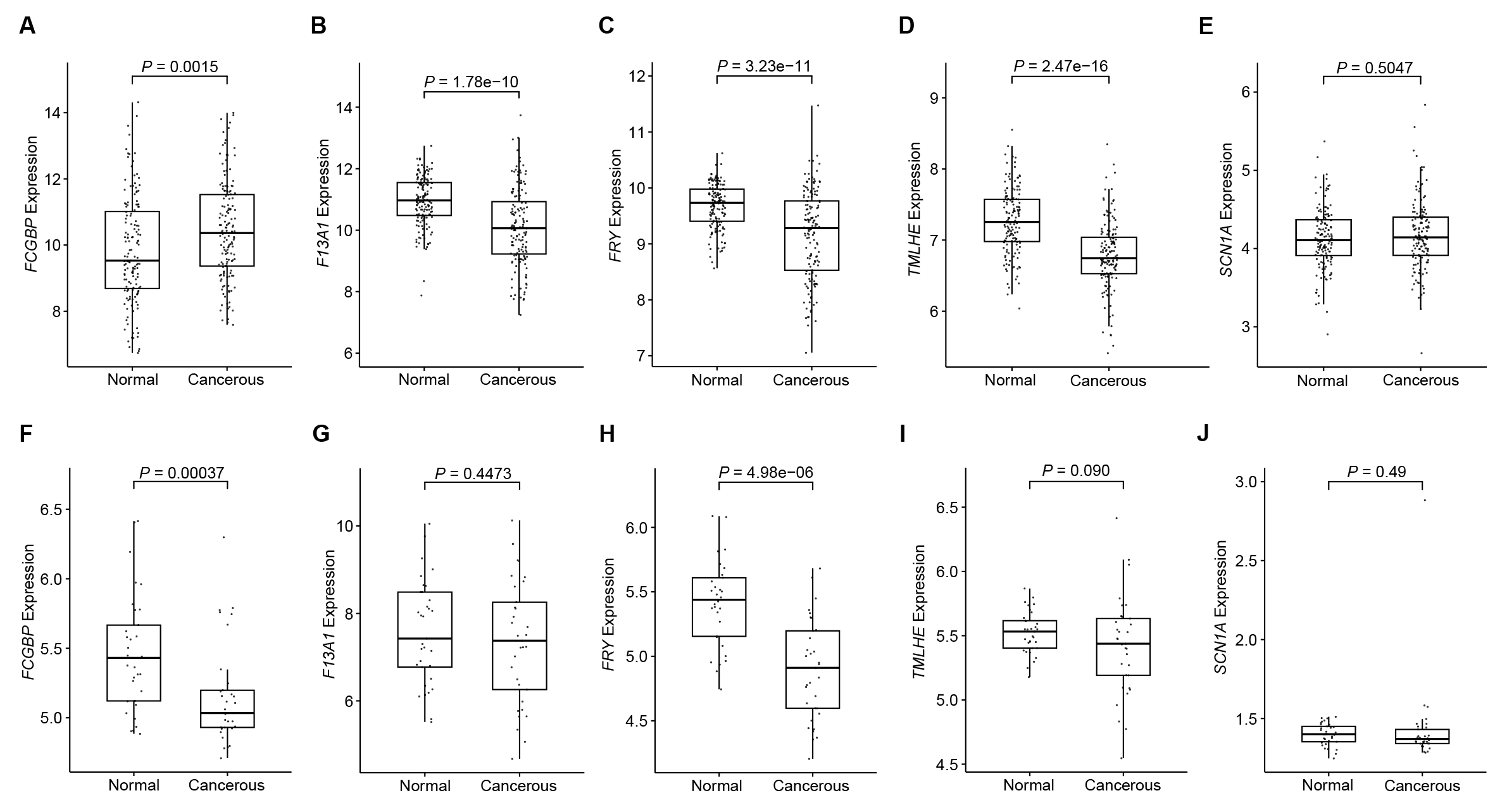
**

**Fig. S5. Microarray expression levels of the five genes in normal and cancerous breast tissues.** **A-E** Comparison of breast cancer risk gene expression in *FCGBP*, *F13A1*, *FRY*, *TMLHE* and *SCN1A*, sourced from dataset E-GEOD-70951. **F-J** Comparison of breast cancer risk gene expression in *FCGBP*, *F13A1*, *FRY*, *TMLHE* and *SCN1A*, sourced from dataset E-GEOD-76250. *P* values were from Wilcoxon signed rank test.

**
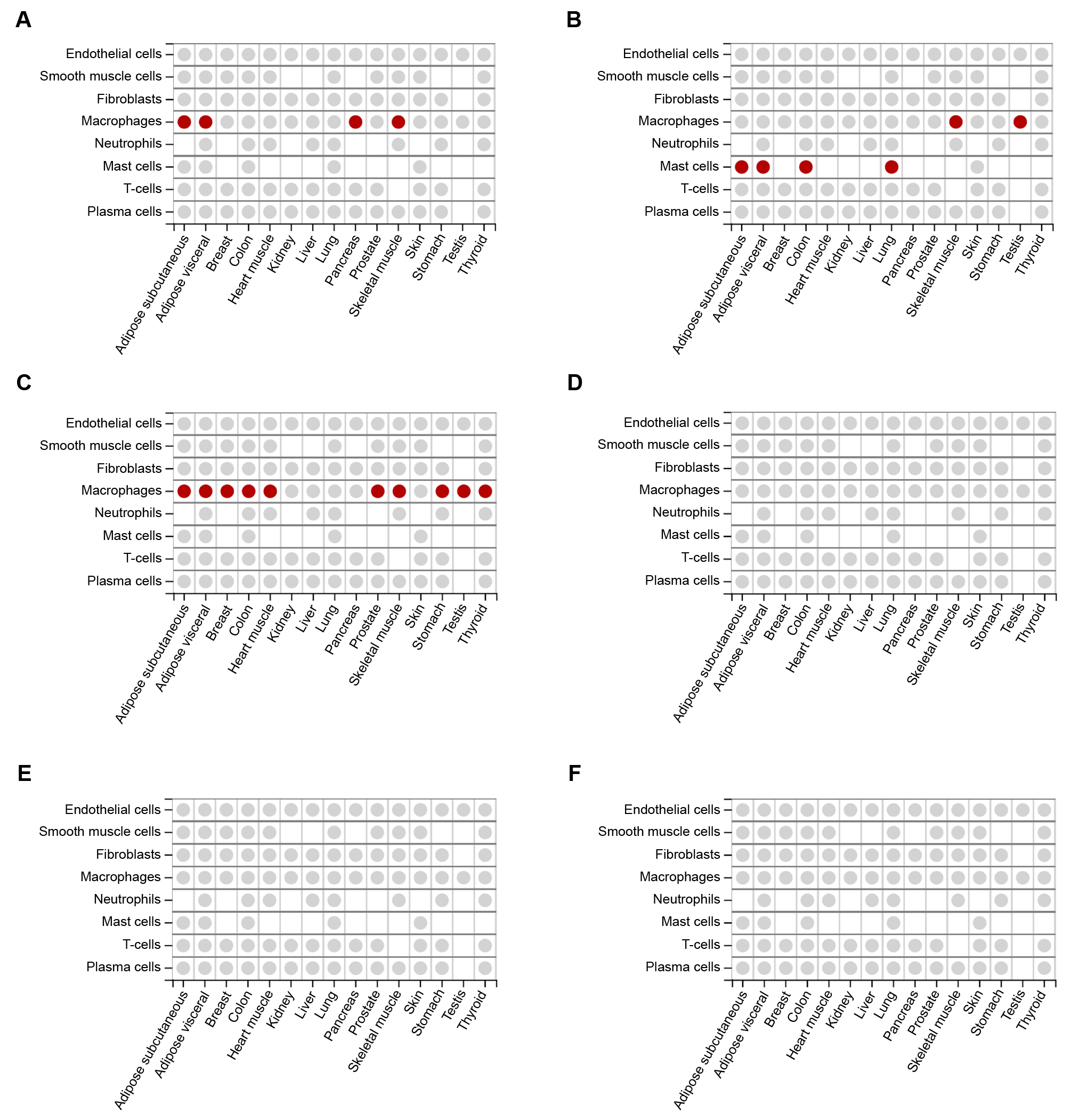
**

**Fig. S6.** **Core cell types associated with the six genes in tissues, sourced from the Human Protein Atlas.** **A** *FCGBP*, **B** *TPSB2*, **C** *F13A1*, **D** *FRY*, **E** *TMLHE*, **F** *SCN1A*. Red dots indicate that the gene has core cell type specificity in the indicated tissue. Grey dots indicate that cell type is present within that tissue, but the selected gene is not predicted to be enriched there.


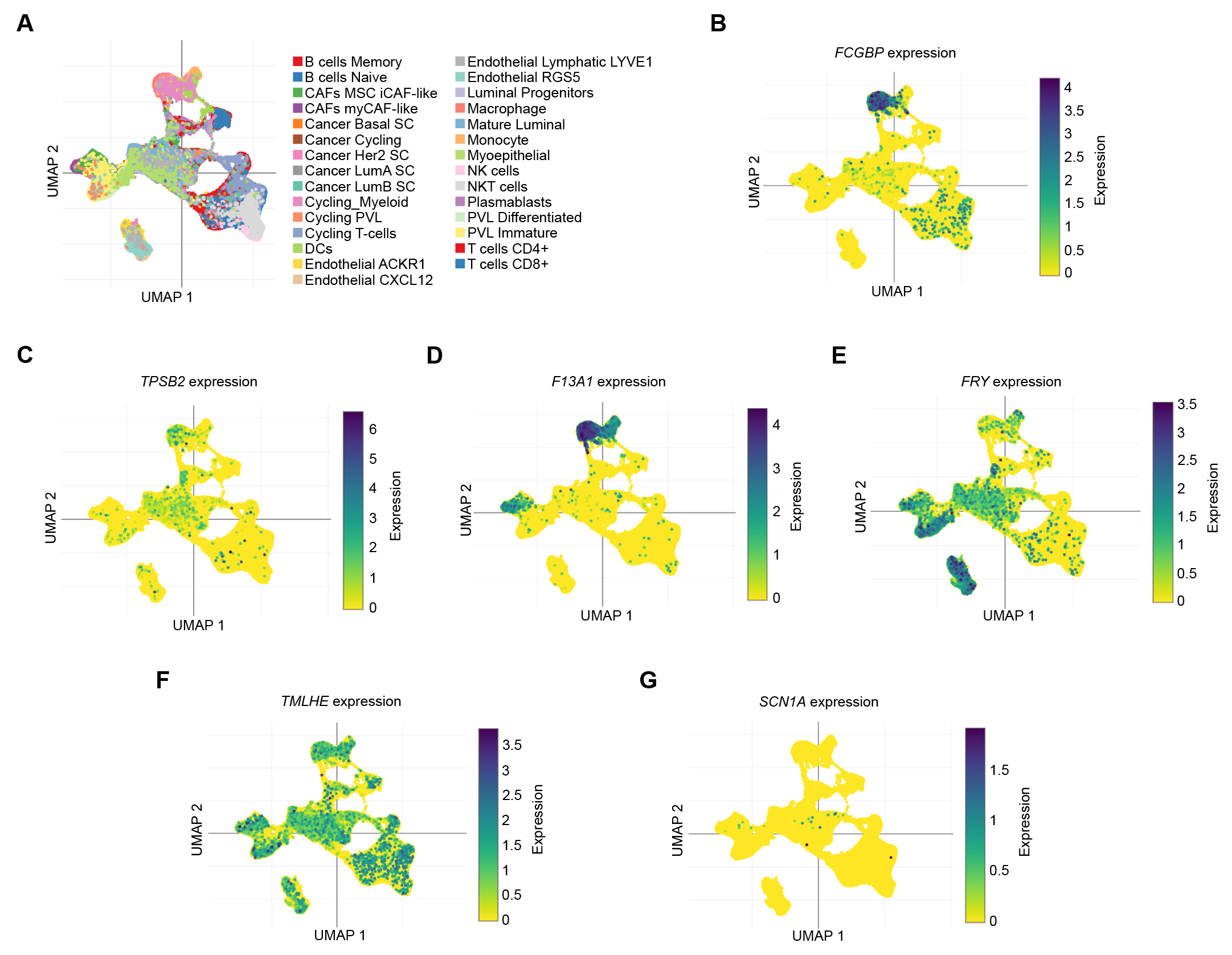


**Fig. S7.** **single-cell RNA expression levels of the six genes in breast tissues, sourced from the Breast Cancer Atlas of Single Cell portal.** **A** UMAP visualization of single cells, colored by cell type. **B-G** Expression of *FCGBP*, *TPSB2*, *F13A1*, *FRY*, *TMLHE* and *SCN1A*, respectively.
